# Kidney Cyst Epithelia in Tuberous Sclerosis Complex (TSC) Exhibit Propagation and Expansion of A-Intercalated, but Loss of B-Intercalated and Principal Cells: The Role of FOXI1, FOXP1, and DMRT2 Transcription Factors

**DOI:** 10.3390/ijms27177515

**Published:** 2026-08-22

**Authors:** Manoocher Soleimani, Kamyar Zahedi, Marybeth Brooks, Julie G. In, Sharon Barone

**Affiliations:** 1 Research Services, New Mexico Veterans Health Care System, Albuquerque, NM 87108, USA; kzahedi@salud.unm.edu (K.Z.); marbrooks@salud.unm.edu (M.B.); sbarone@salud.unm.edu (S.B.); 2 Department of Internal Medicine, University of New Mexico School of Medicine, Albuquerque, NM 87106, USA; jgin@salud.unm.edu

**Keywords:** tuberous sclerosis complex, A-Intercalated cells, DMRT2, FOXP1, FOXI1, cystogenesis, kidney, transcription factors

## Abstract

Kidney cystic epithelium primarily comprises proliferating A-intercalated (A-IC) cells in humans and mouse models of TSC. These studies explored the expression of transcription factors (TF) that drive the development of A-IC cells and the downregulation of B-intercalated (B-IC) cells, as well as the status and localization of *Tsc1* and *Tsc2* in epithelial cells lining the cysts. Transcriptome studies indicated enhanced expression of the following TFs: DMRT2, FOXI1, and FOXP1 in young TSC mice, and DMRT2 and FOXI1 in aged mice. The expression of *Hmx2* decreased in TSC mice of all ages. Our studies further demonstrated: (1) distinct and predominant DMRT2 and FOXP1 localization in the cystic epithelium of in TSC mouse models; and (2) expression of *Foxi1*, *Tsc1*, and *Tsc2* in epithelial cells lining the kidney cysts. Expression of DMRT2, FOXI1, and FOXP1 is enhanced in the A-IC cells lining the kidney cysts of TSC mouse models. In the same models, the expression of *Hmx2* mRNA is decreased and is accompanied by the loss of B-IC cells in cyst epithelia. Both DMRT2 and FOXP1 localized to the nucleus of A-IC cells of the renal cysts, suggesting that TSC kidney cystogenesis is driven by factors that exclusively promote A-IC expansion.

## 1. Introduction

Tuberous sclerosis complex (TSC) is an autosomal dominant disease caused by mutations that affect the hamartin (TSC1) and tuberin (TSC2) proteins [1,2,3]. These proteins are part of a trimolecular complex that regulates the activity of the mechanistic target of rapamycin complex 1 (mTORC1), thereby, controlling cell growth and proliferation [4,5]. Nearly 2 million people are estimated to be living with TSC [3]. TSC affects multiple organs, including the kidney, lung, heart, and brain [3,6]. Close to 85% of individuals afflicted with TSC present with renal cysts and angiomyolipomata (AML) [7,8], which can eventually lead to kidney failure [7,8,9]. The factors that promote the development of renal lesions (e.g., cysts and AML) are poorly understood.

Examination of kidney cysts in individuals with TSC and in mouse models of TSC indicates that these lesions primarily comprise proliferating A-intercalated (A-IC) cells with intact TSC1 and TSC2 [10,11], suggesting that the mechanism of renal cystogenesis is not concordant with the genetic explanation of TSC. The dominance of A-IC cells lining the kidney cysts is a unique phenotype that is detected in the global inactivation of *Tsc2* (*Tsc2^+/−^* mice) and in the inactivation of *Tsc1* or *Tsc2* in principal cells (PC), in *Tsc1* inactivation in pericytes, as well as in renal cyst biopsies from individuals with TSC [10,11]. This cellular phenotype is distinct from the epithelial cells lining the kidney cysts in *Pkd* mutant mice or in individuals with Autosomal Dominant Polycystic Kidney Disease (ADPKD), which are predominantly composed of the PC [10,12].

Recent studies indicated that Forkhead Box P1 (FOXP1), a member of the Forkhead Box (FOX) family of transcription factors, may play a crucial role in cellular development and function across various tissues [13,14,15,16]. In the kidney collecting duct, FOXP1 is thought to regulate the expression of two transcription factors, DMRT2 and HMX2 [16]. DMRT2 belongs to the family of Doublesex and Mab-3-related transcription factors (DMRT2) and is involved in various developmental processes [17,18,19]. In the kidney collecting duct, DMRT2 is presumed to promote the development of A-IC cells [20,21]. HMX2 belongs to the NKL homeobox family, which is known for its role in organ development [22]. In the kidney collecting duct, HMX2 is critical for the development of B-intercalated cells (B-IC) [20,21]. FOXP1 has been implicated in the transcriptional regulation of both the *Dmrt2* and *Hmx2* genes [16].

Notch-mediated cell–cell interactions play a crucial role in maintaining cell division and the subsequent generation of cell-type diversity in various organs [23,24]. In the kidney collecting duct, Notch signaling has been shown to inhibit the conversion of PC to A-IC and B-IC cells by activating HES1 [25,26]. These studies have further indicated that the suppression of HES1 activates the de-differentiation of PC to A-IC and B-IC cells [24,25,26].

Published studies have shown that a network of transcription factors composed of FOXI1, FOXP1, DMRT2 and HMX2 regulate the development and differentiation of intercalated cells [16,20,21,27]. However, the role of this network of transcription factors in the development of TSC renal cysts has not been examined. The purpose of the current study was to examine the expression, localization, and association of transcription factors FOXP1, DMRT2, and HMX2 in kidney cyst development in TSC mouse models. To this end, RNA-seq analyses were performed on the kidneys of various TSC mouse models and confirmed by appropriate Western blot analyses as well as immunohistochemical, immunofluorescence and fluorescence in situ hybridization (FISH) microscopic studies.

## 2. Results

### 2.1. RNA Transcriptome Analysis of Dmrt2, Foxi1, Foxp1, and Hmx2 in Kidney Cyst Epithelia in TSC

**Figure 1:**
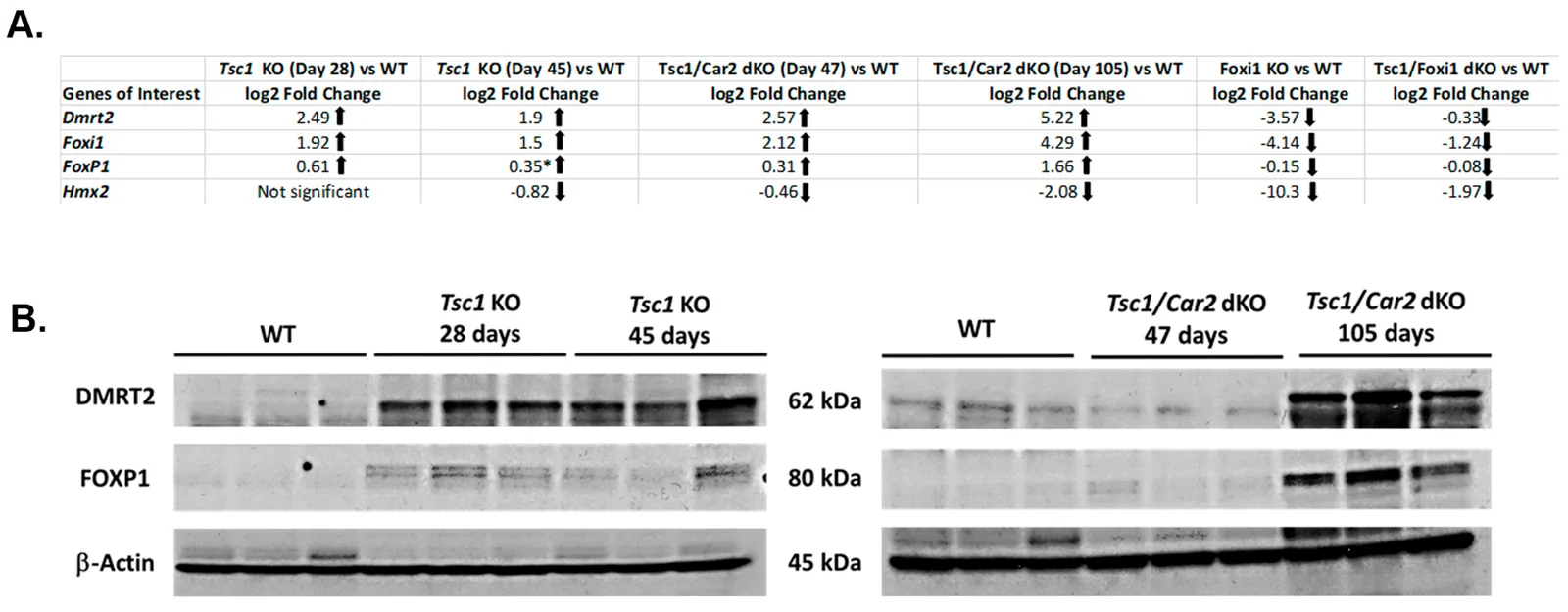
RNA-seq and western blot analyses comparing the renal transcriptomes of various TSC mouse models. (**A**) Tabulated presentation of RNA-seq results comparing young and old *Tsc1* KO (28-day-old and 45-day-old) and *Tsc1/Car2* dKO (47-day-old and 105-day-old) to WT mice. *Foxi1* KO and *Tsc1/Foxi1* dKO mice (with no cyst presentation) are also included. All RNA-seq analysis were performed using n = 3 animals/genotype/timepoint. Black arrows indicate if the renal transcriptome was upregulated or downregulated. * For *Tsc1* KO 45-day-old the *p* value was significant (*p* = 0.044), but the adjusted *p* value (*p* = 0.313) was not (n = 3/strain) (**B**) Left panels show the Western blot images of FOXP1 and DMRT2 levels in the kidneys of 28 day (low cyst burden) and 45-day-old *Tsc1* KO (moderate cyst burden). Right panels show the Western blot images of FOXP1 and DMRT2 levels in the kidneys of 47-day-old (low cyst burden) and 105-day-old *Tsc1/Car2* dKO (heavy cyst burden) mice (n = 3/group/timepoint).

The expression of Forkhead Box I1 (*Foxi1*) and *Dmrt2* increased significantly in the kidneys of both young *Tsc* mice (*Tsc1* KO; 28-day-old) and older *Tsc* mice (*Tsc1* KO; 47-day-old). Alternatively, the expression of Forkhead Box P1 (*Foxp1*) was significantly elevated in 28-day-old (log_2_ fold change = 0.61; adj *p* = 0.0023), but not in 45-day-old (log_2_ fold change = 0.35; adj *p* = 0.313) *Tsc1* KO mice when compared to their WT counterparts (Figure 1A). The above mRNAs were also upregulated in 47- and 105-day-old *Tsc1/Car2* double knockout (*Tsc1/Car2* dKO) mice but were significantly downregulated in the kidneys of *Tsc1/Foxi1* double knockout (*Tsc1/Foxi1* dKO; no cysts), and *Foxi1* KO mice. The expression of the *Hmx2* transcription factor mRNA decreased significantly in the kidneys of 45-day-old *Tsc1* KO (log_2_ fold = −0.82; adj *p* = 0.099) and 105-day-old *Tsc1/Car2* dKO (log_2_ fold = −2.08; adj *p* = 3.24 × 10^−5^) mice, where both models exhibit large and numerous A-IC lined kidney cysts (Figure 1A). In the next series of studies, Western blot analyses were performed to determine the expression of FOXP1 and DMRT2 protein levels in *Tsc1*KO (moderate cyst burden) and *Tsc1/Car2* dKO mice (heavy cyst burden; Figure 1B). As shown, the results of our Western blot analyses (Figure 1B) confirm our RNA-Seq results (Figure 1A).

### 2.2. Cells Lining the TSC Renal Cysts Are Predominantly Proliferatively Active A-IC Cells That Express Both Tsc1 and Tsc2

**Figure 2:**
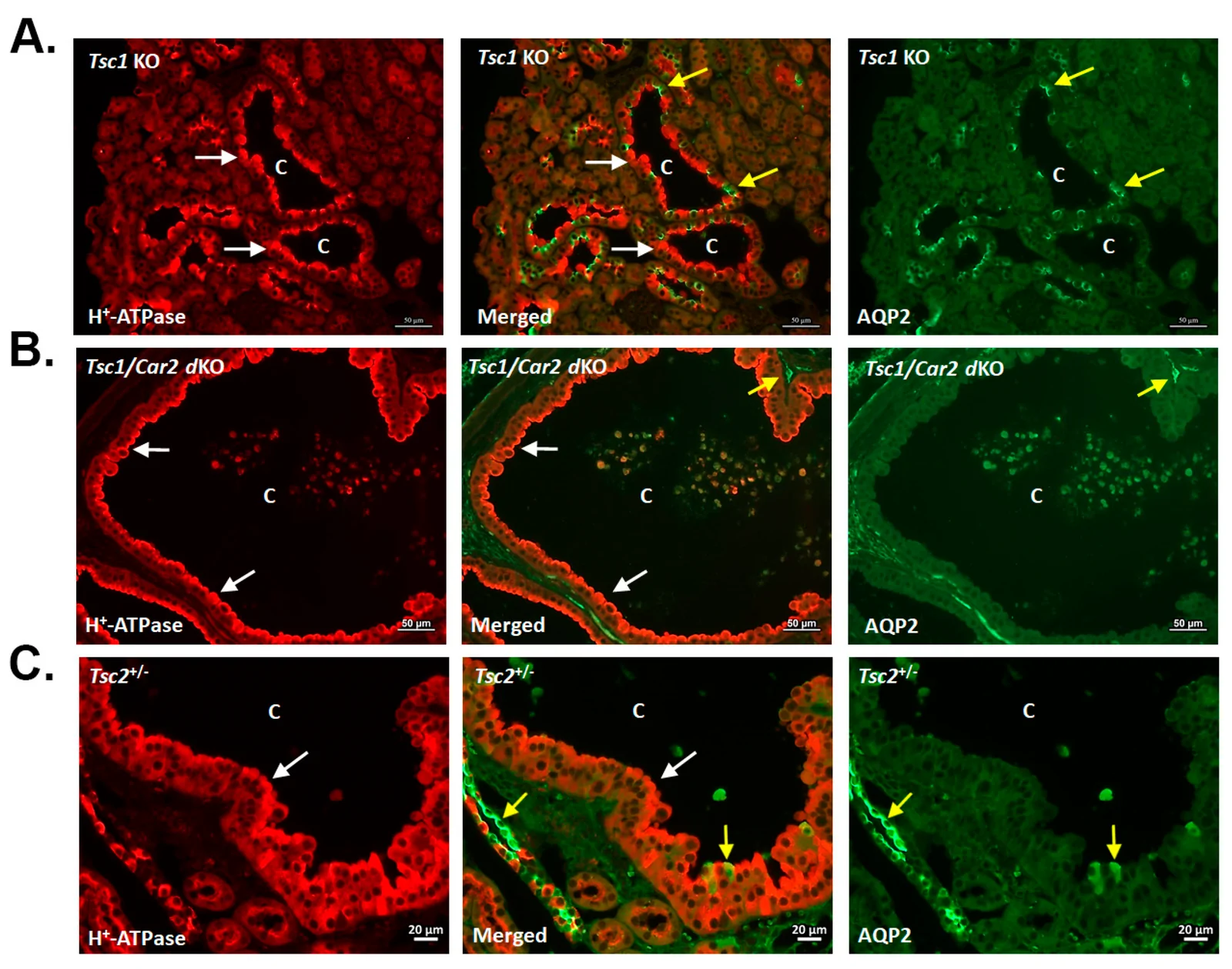
Localization and expression of H^+^-ATPase and AQP2 in the cystic epithelium of various TSC mouse models. Double-labeling of H^+^-ATPase (left panel; white arrows) and AQP2 (right panel; yellow arrows) lining the cysts in 47-day-old *Tsc1* KO (**A**), 105-day-old *Tsc1/Car2* dKO (**B**), and 300-day-old *Tsc2^+/−^* (**C**) mice. The middle panel depict the merged images. “C” represents cysts. Scale bar for (**A**) is 50 μm, (**B**) is 50 μm, and (**C**) is 20 μm. Images are representative of those observed in n = 3 mice.

In the next series of studies, we verified the identity and proliferative status of cells lining the kidney cysts in TSC mouse models. Figure 2A illustrates double-labeling with H^+^-ATPase, a marker of A-IC cells, and AQP2, a marker of PC, in the kidneys of mice with *Tsc1* inactivation in PC (*Tsc1* KO). As shown, cells lining the cysts were predominantly A-IC cells, as verified by the expression of H^+^-ATPase on their apical domain. Very few cells lining the cysts expressed AQP2, indicating a scarcity of principal cells (PC). As we previously reported, the expression of B-IC cells, as assessed and examined by the localization of pendrin, was also greatly diminished in the renal cystic epithelia of various mouse models of TSC [10,11]. The examination of the severely cystic kidneys of 105-day-old *Tsc1/Car2* dKO mice (Figure 2B) demonstrated a similar cellular phenotype, in which the epithelial lining of the cysts almost entirely comprised cells that expressed apical H^+^-ATPase, with few cells showing AQP2 staining. Double immunostaining of the kidneys of 450-day-old *Tsc2^+/−^* mice (Figure 2C), which present with numerous cysts and cystadenomas, with H^+^-ATPase and AQP2 antibodies also revealed the preponderance of A-IC cells (H^+^-ATPase positive) and a paucity of B-IC cells in the cystic epithelium.

**Figure 3:**
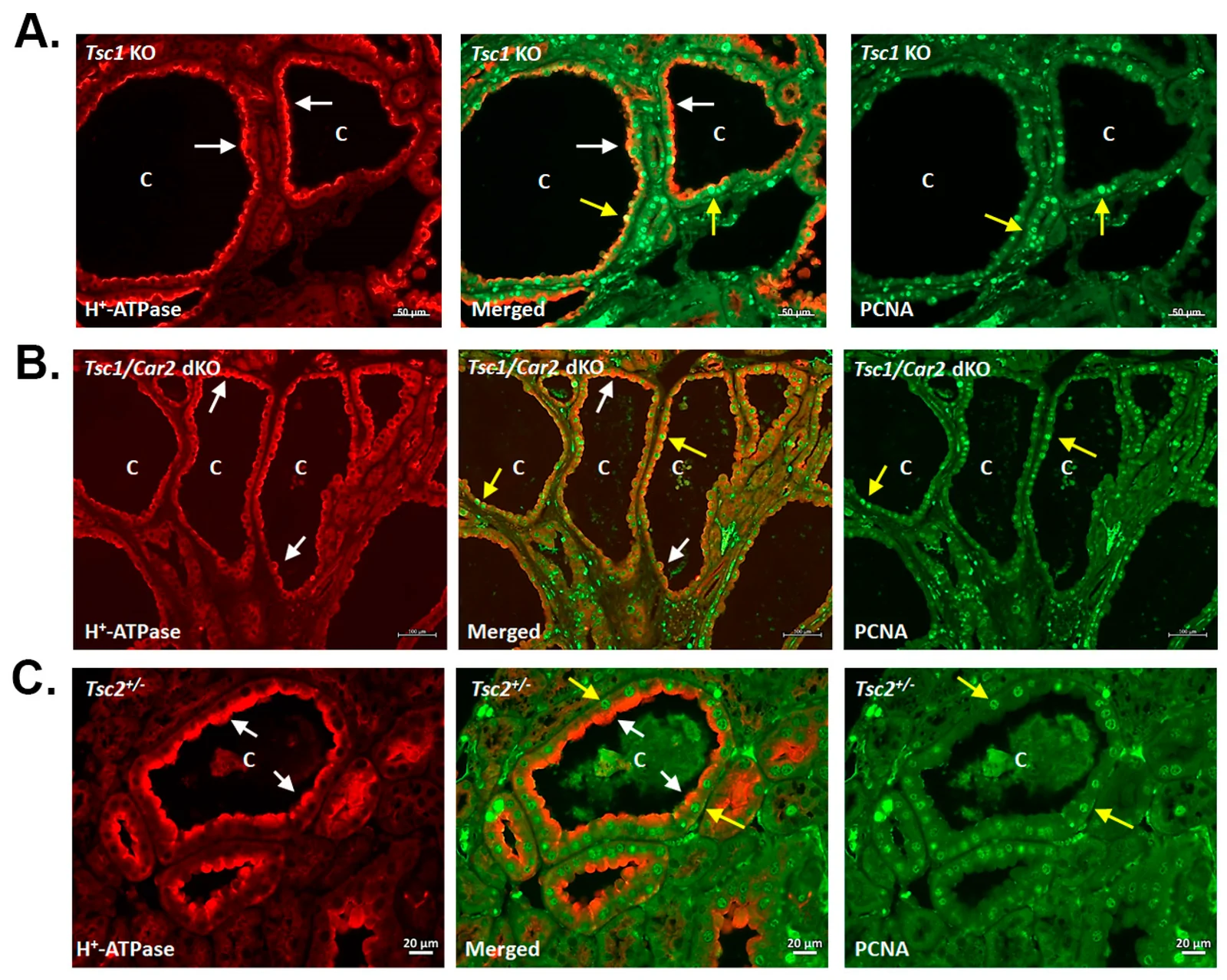
Cell proliferation in epithelial cells lining the cysts in different TSC mouse models. Immunofluorescence double-labeling with H^+^-ATPase (white arrows; left panel) and PCNA (right panel; yellow arrows) in 47-day-old *Tsc1* KO (**A**), 105-day-old *Tsc1/Car2* dKO (**B**), and 300-day-old *Tsc2^+/−^* (**C**) mice. PCNA labeling indicates active cellular proliferation that is occurring in epithelial cells lining the cysts. “C” represents cysts. Scale bar for (**A**) is 50 μm, (**B**) is 100 μm, and (**C**) is 20 μm. Images are representative of those observed in n = 3 mice.

Next, we examined the proliferative status of the cystic epithelial cells. Figure 3A depicts double-labeling with H^+^-ATPase, a marker of A-IC cells, and Proliferating Cell Nuclear Antigen (PCNA), a marker of cell proliferation, in the kidneys of *Tsc1* KO mice. As shown, cells lining the cysts overwhelmingly expressed PCNA in their nuclei, along with H^+^-ATPase on their apical domain, consistent with hyperproliferative A-IC cells.

Figure 3B,C illustrate a similar double immunofluorescence staining pattern in the kidneys of *Tsc1/Car2* dKO and *Tsc2^+/−^* mice, respectively. As indicated, cells lining the kidney cysts in these mouse models show significant, widespread intracellular PCNA localization, along with apical H^+^-ATPase localization. An additional high-power magnification image showing nuclear localization of PCNA in the renal cystic epithelium of *Tsc1* KO mice is included in Supplement Figure S1A.

**Figure 4:**
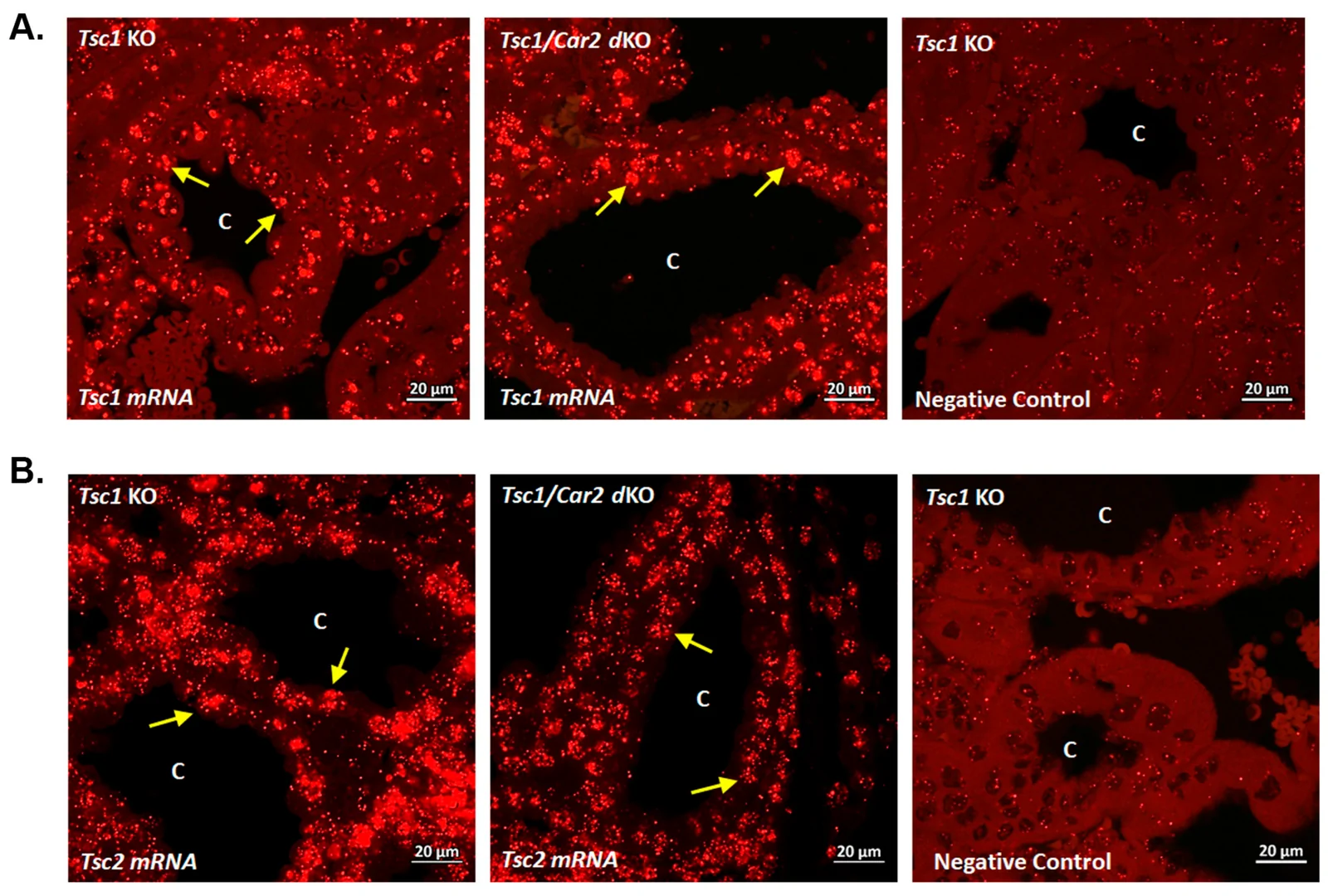
Tsc1 and Tsc2 mRNA expression in epithelial cells lining the cysts in various TSC mouse models. Utilizing fluorescence in situ hybridization (FISH), the localization of *Tsc1* (**A**) and *Tsc2* (**B**) mRNA (yellow arrows) is shown in the epithelial cells lining the cysts in 47-day-old *Tsc1* KO (left panels in (**A**,**B**)) and 105-day-old *Tsc1/Car2* dKO mice (middle panels in (**A**,**B**)). Negative control in *Tsc1* KO mice (right panel in (**A**,**B**)) shows little non-specific signal. “C” represents cysts. Scale bar = 20 μm. Images are representative of those observed in n = 3 mice.

The primary mouse model of TSC renal cystogenesis in these studies, *Tsc1* KO, requires specific ablation of the *Tsc1* gene in PC; however, the cystic epithelium in the kidneys of these mice is primarily composed of A-IC cells. Therefore, we examined the mRNA expression of both the *Tsc1* and *Tsc2* in the renal cysts in *Tsc1* KO and *Tsc1/Car2* dKO mice (Figure 4A,B). Our fluorescence in situ hybridization (FISH) results demonstrated strong expression and localization of both *Tsc1* and *Tsc2* transcripts to the epithelial lining of the TSC kidney cysts (Figure 4). These results indicate that the renal cystic epithelial cells are primarily composed of actively dividing A-IC cells that express both *Tsc1* and *Tsc2* transcripts.

### 2.3. The Expression of Factors That Drive A-IC Cell Development Is Enhanced in the Epithelium of Renal Cysts in Mouse Models of TSC

**Figure 5:**
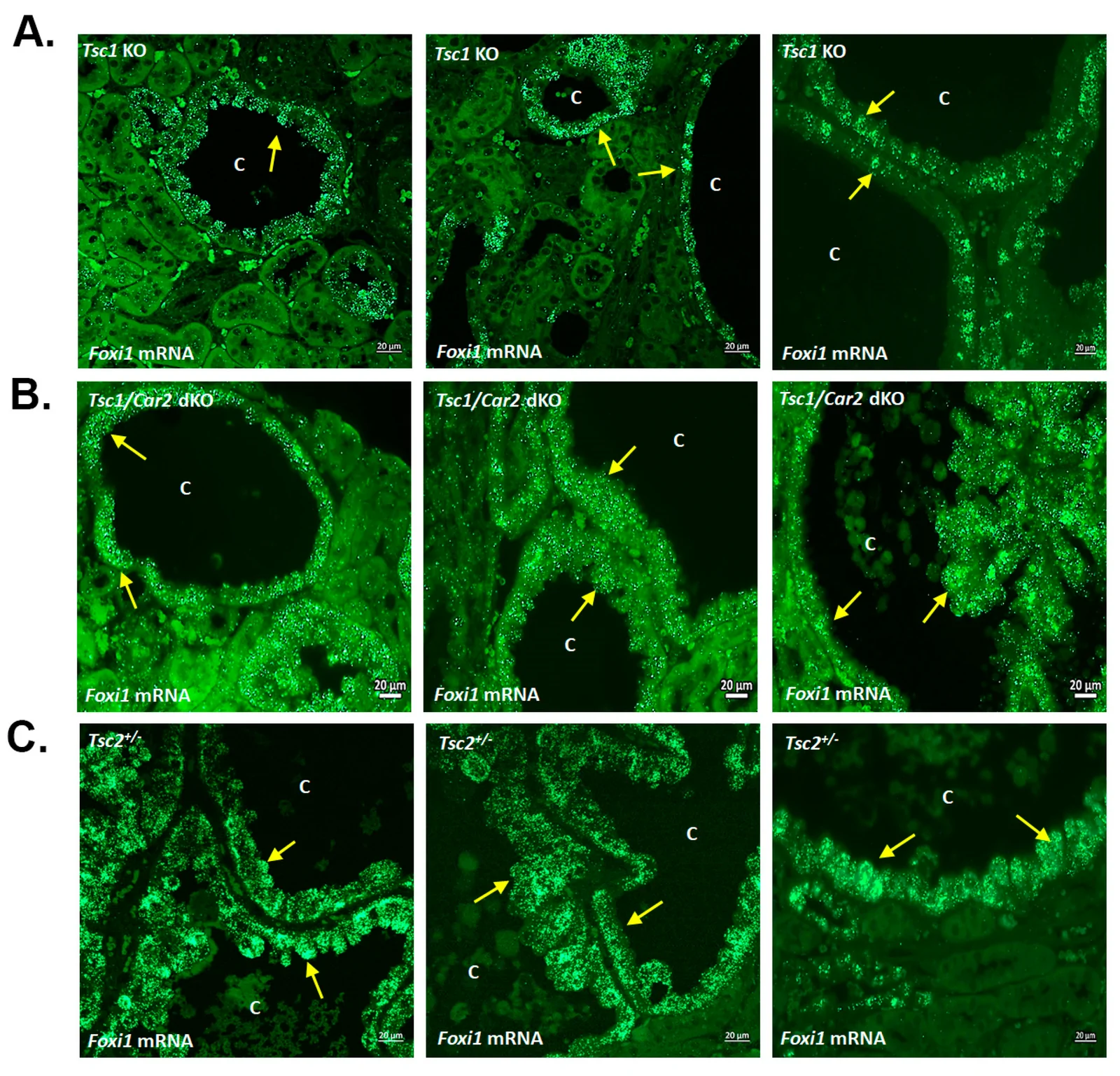
Foxi1 mRNA expression in epithelial cells lining the cysts in various TSC mouse models. Utilizing FISH, *Foxi1* mRNA localization (yellow arrows) is shown in the epithelial cells lining the cysts in 47-day-old *Tsc1* KO ((**A**); n = 3), 105-day-old *Tsc1/Car2* dKO ((**B**); n = 3), and 300-day-old *Tsc2^+/−^* ((**C**); n = 3) mice. Three images from each strain are presented to show Foxi1 expression in multiple mice. “C” represents cysts. Scale bar = 20 μm.

Based on our RNA-seq studies, we examined the expression of factors involved in A-IC cell development in mouse models of TSC. The expression of *Foxi1* mRNA in the kidneys of *Tsc1* KO, *Tsc1/Car2* dKO, and *Tsc2^+/−^* mice was examined by FISH. We employed this approach in place of our immunostaining using various anti-FOXI1 antibodies, which failed to yield specific labeling. As indicated in Figure 5A–C, *Foxi1* shows strong and almost universal expression in the cystic epithelial cells.

**Figure 6:**
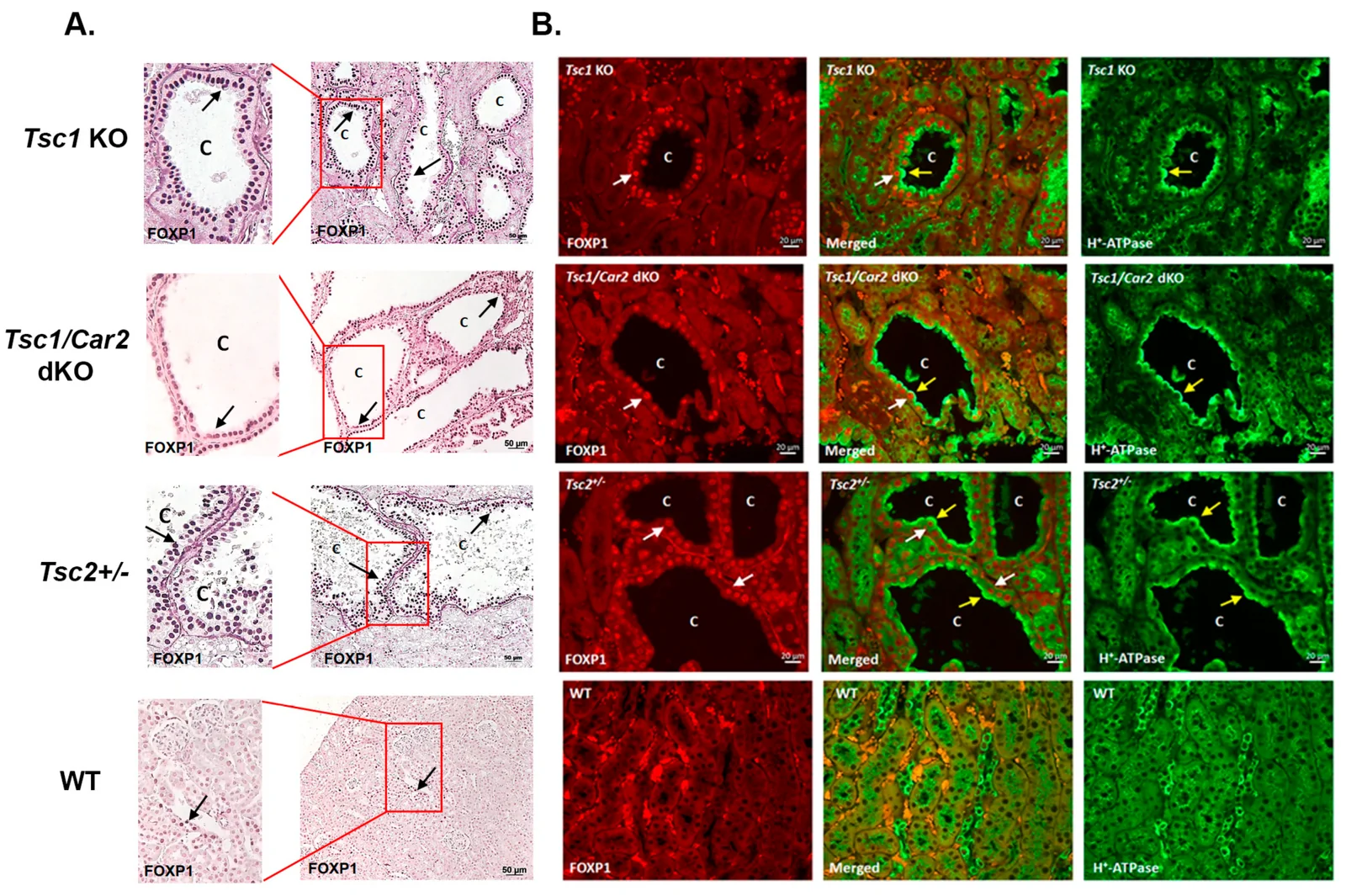
FOXP1 expression and localization in epithelial cells lining the cysts in various TSC mouse models. (**A**) FOXP1 (black arrows) can be found in the nucleus of the epithelial cells lining the cysts in 47-day-old *Tsc1 KO* (left and right panels of the first row), 105-day-old *Tsc1/Car2 dKO* (left and right panels of the second row), and 300-day-old *Tsc2^+/−^* (left and right panels of the third row) mice. These were compared to the localization of FOXP1 in WT mice (left and right panels of the fourth row). Both high (left) and low (right) magnifications are shown. “C” represents cysts. Scale bar = 50 μm. (**B**) Immunofluorescence double-labeling of FOXP1 (left panels; white arrows) and H^+^-ATPase (right panels; yellow arrows) in the epithelial cells lining the cysts in various mouse models of TSC. All TSC mouse models (*Tsc1* KO, *Tsc1/Car2* dKO, and *Tsc2^+/−^*) show similar expression and localization of FOXP1 and H^+^-ATPase in their cystic epithelium. FOXP1 and H^+^-ATPase expression was compared to WT mice (bottom panel). “C” represents cysts. Scale bar = 20 μm. Images are representative of those observed in n = 3 mice.

Next, we examined the expression of FOXP1, another protein that belongs to the Forkhead Box family of TFs, which acts in tandem with FOXI1 and is important in the differentiation and determination of A-IC and B-IC cell fate [16]. Figure 6 demonstrates the expression of FOXP1 in the kidneys of *Tsc1* KO mice at 45 days of age, *Tsc1/Car2* dKO mice at 105 days of age (numerous large cysts), and *Tsc2^+/−^* mice at 450 days of age (numerous large cysts) by immunohistochemical and immunofluorescence labeling. Immunohistochemical localization of FOXP1 in the kidneys of TSC mouse models (Figure 6A) shows its strong, widespread nuclear expression in cystic epithelial cells. The expression of FOXP1, while nuclear, is significantly less intense in the kidneys of WT mice (Figure 6A, fourth panel). Additional immunofluorescence microscopy studies using FOXP1 and H^+^-ATPase antibodies established that FOXP1-positive cystic cells also expressed H^+^-ATPase on their apical membrane (Figure 6B). An additional high-power magnification image showing nuclear localization of FOXP1 in *Tsc1* KO mice is included in Supplement Figure S1B. These results provide further evidence that the TF network driving intercalated cell differentiation is active in A-IC cells lining the epithelium of renal cysts in TSC mouse models.

**Figure 7:**
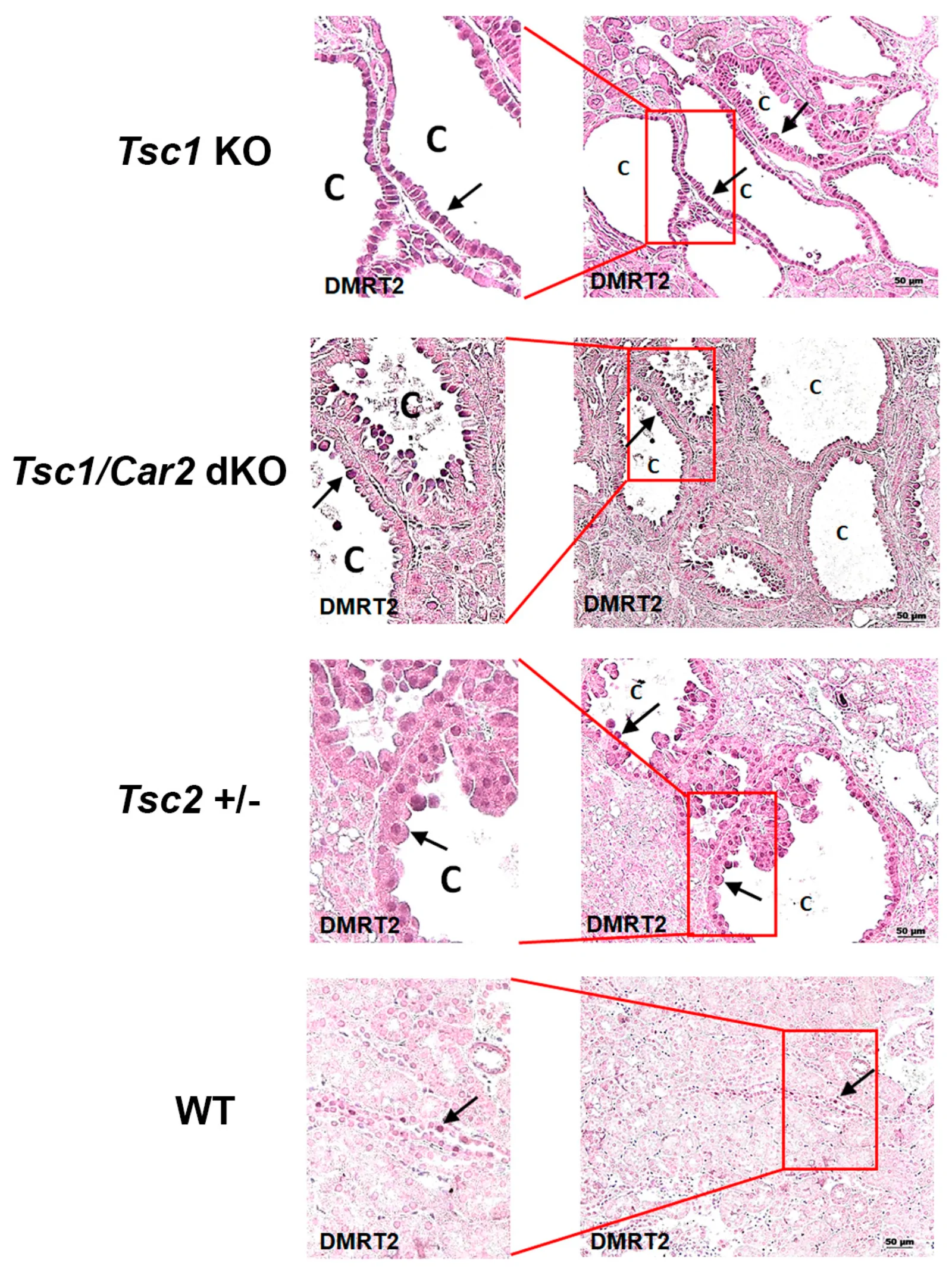
DMRT2 expression and localization in epithelial cells lining the cysts in various TSC mouse models. Localization of DMRT2 (black arrows) is demonstrated in the nucleus of the epithelial cells lining the cysts in 47-day-old *Tsc1 KO* (**left** and **right** panels of the first row), 105-day-old *Tsc1/Car2 dKO* (**left** and **right** panels of the second row), and 300-day-old *Tsc2^+/−^* (**left** and **right** panels of the third row) mice. These were compared to localization in WT mice (**left** and **right** panels of the fourth row). Both high (**right**) and low (**left**) magnifications are shown. “C” represents cysts. Scale bar = 50 μm. Images are representative of those observed in n = 3 mice.

Increased expression of DMRT2 drives the differentiation of A-IC cells [16,20,21]. Therefore, we examined the expression and localization of this TF in the kidneys of mouse models of TSC. Figure 7 and Supplement Figure S1C illustrate the immunohistochemical localization of DMRT2 in the kidney cysts of TSC mouse models, including *Tsc1* KO, *Tsc1/Car2* dKO, and *Tsc2^+/−^* mice. The results demonstrate that the cells lining the cyst epithelia in the kidneys of these mice show widespread expression and nuclear localization of DMRT2.

## 3. Discussion

Several models of mouse TSC disorder were utilized in the current study to examine the expression and localization of transcription factors FOXP1, FOXI1 and DMRT2 in TSC renal cysts. These models included *Tsc1* KO, *Tsc1/Car2* dKO, *Tsc1/Foxi1* dKO and *Tsc2^+/−^* mice. The models used in these studies, especially the various modifications of *Tsc1* KO mice, cause cysts of various severities and have previously been used to examine the process of renal cystogenesis in TSC [10,11,28]. The primary reason for using these mouse models is that their cysts closely mimic those in human TSC [10,11,28]. Specifically, the cystic epithelium in human and mouse models does not show “loss of heterozygosity” (the primary driver of angiomyolipomata development), exhibits expansion of A-IC cells in the cyst epithelium, and shows increased expression of A-IC-associated markers [10,11,12].

The hyperproliferation of A-IC cells lining the kidney cysts is a phenomenon unique to TSC and distinct from cysts found in ADPKD, which originate from the collecting duct PC [10,12]. Mice with kidney PC- or pericyte-specific ablation of *Tsc1 or Tsc2* genes, as well as *Tsc2^+/−^* mice, develop numerous cortical kidney cysts, which primarily comprise proliferating A-IC cells [10,11]. The preponderance of A-IC cells in renal cyst epithelium has also been documented in kidney tissues of TSC patients [10,11,28,29].

The robust presence of A-IC cells in cyst epithelium was also evident in tamoxifen-induced *Tsc1* KO in PC (^ECE/+^*Tsc1-KO*) in adult mice [28]. Our results unequivocally demonstrate the critical role of A-IC cells in TSC cystogenesis. They further indicate that molecules associated with A-IC cell proliferation and viability (e.g., FOXI1, c-KIT) play key roles in TSC renal cystogenesis [10,28]. These results suggest a common kidney cystogenic mechanism in TSC that depends on the expansion of A-IC cells. In contrast, kidney cysts in ADPKD predominantly comprise PC, not A-IC cells [12,30].

Activated mTORC1 is the main driver of tumor growth and cystogenesis in TSC [8,9,31]. This is evidenced by the increased levels of its activation markers, phosphorylated ribosomal protein S6 kinase (S6K) and phosphorylated ribosomal S6 protein (pS6), in TSC-associated lesions [10,11,32]. In mammals, mTORC1 is a protein complex that acts as an integrator of signals, responding to growth factors and sensing the availability of nutrients (e.g., amino acids); thereby, regulating cell growth and proliferation [33]. Published reports indicate that mTORC1 is highly active in both tuberous sclerosis complex (TSC) and Autosomal Dominant Polycystic Kidney Disease (ADPKD) and is a well-documented driver of kidney lesions and cyst development in both disease states. This is noteworthy because, despite mTORC1 activation, the epithelial cells lining the kidney cysts in TSC and ADPKD express a phenotypically distinct cell lineage that lines their respective cysts [10,12]. Furthermore, while the epithelial lining of the kidney cysts in TSC is predominantly composed of hyperproliferating Type A intercalated cells (A-ICs)**,** the cystic epithelial cells in ADPKD lack A-ICs and are instead lined by de-differentiated principal cells and other tubule lineages [12]. Under normal conditions, the TSC protein complex, which comprises TSC1, TSC2 and TBC1D7, modulates the activity of mTORC1 in response to the growth environment [4,5]. Whereas mTORC1 activity, because of its physiological role (integration of environmental signals and cell growth), is detected in all tissues, including the kidney of unaffected individuals and WT animals (illustrated by the presence of pS6), its activity is significantly elevated in TSC-associated lesions in various tissues of all individuals with TSC and all TSC mouse models [8,34]. Related to the abundant presence of principal cells lining the kidney cysts in ADPKD, tolvaptan, an AVP2R antagonist, is currently used as the primary pharmaceutical option for the treatment of ADPKD [35,36,37,38]. However, there are no known therapeutically targetable molecules that can treat cysts and other lesions in the TSC-affected kidney, except for rapalogs, whose effects are transient [31,39].

Utilizing RNA sequencing (RNA-Seq) and expression studies, we identified a robust expression of FOXI1 and its downstream molecules, including apical H^+^-ATPase and cytoplasmic carbonic anhydrase 2 (CAR2), in cyst epithelia of *Tsc1*cKO/*Aqp2*cre (*Tsc1* KO) and *Tsc2*cKO/*Aqp2*cre (*Tsc2* KO) mice [10,32]. The ablation of the *Foxi1* gene, which is crucial for H^+^-ATPase expression and intercalated cell development [27], in *Tsc1*KO mice inhibited mTORC1 activation and prevented renal cyst formation [10]. In *Tsc1* KO mice, inactivation of the *Car2* gene, which is necessary for intercalated cell development, also significantly reduced cyst size and delayed the development of renal cysts [32]. Our recent studies demonstrate that proto-oncogene c-KIT, a resident molecule in A-IC cells, which is highly expressed in the cyst epithelium and is regulated by FOXI1 drives kidney cystogenesis in *Tsc1* KO mice [28].

The preponderance of A-IC cells lining the cyst epithelia in TSC is associated with the absence of B-IC cells [10,11]. This unique observation strongly suggests the activation of pathways that favor the expansion and preponderance of only A-IC cells in cyst epithelia. A network of TFs comprising FOXI1, FOXP1, DMRT2, and HMX2, regulates the development of intercalated cells and their lineage-specific differentiation into A-IC or B-IC cells [16,20,21,27]. While increased DMRT2 expression drives the differentiation of A-IC cells, HMX2 favors the development of B-IC cells [20,21]. With respect to the mRNA expression of *Foxi1*, our previous studies have consistently demonstrated its enhanced expression in TSC cystic kidneys [10,28]. In addition to FOXI1, which is critical for the development of A-IC and B-IC cells [27], our current studies identify *Foxp1* as a significantly upregulated transcript in TSC mouse models that contain a heavy cyst burden (Figure 5). While published reports indicate that both FOXI1 and FOXP1 play essential roles in kidney collecting duct development by regulating the expression of transcription factors, DMRT2 and HMX2 [20,21,40], our RNA-seq studies demonstrate the upregulation of DMRT2 (Figure 1, Figure 6) and the downregulation of HMX2 (Figure 1). This is in line with published studies indicating that DMRT2 promotes the development of A-IC cells, whereas HMX2 may be critical for the differentiation and propagation of B-IC cells [20,21,40]. The downregulation of HMX2 in our studies is also congruent with the loss of B-IC cells in the cystic epithelia (Figure 1). In support of this, we previously demonstrated that the B-IC cell markers, such as pendrin, are greatly diminished in the epithelia of TSC cysts [10,11].

Our current findings clearly show the localization of FOXP1 to A-IC cells lining the kidney cysts in TSC mouse models based on their co-localization with H^+^-ATPase (Figure 5). These results are congruent with studies using single-cell RNA sequencing and immunostaining, which have shown that *Foxp1* is selectively expressed in intercalated cells within the kidney collecting duct [16]. Whether FOXP1 is merely a marker of intercalated cells or a crucial transcription factor that plays a significant role in intercalated cell development remains to be investigated. Our current studies do not provide unequivocal evidence for the essential role of FOXP1 in intercalated cell development. Whether FOXP1 plays a permissive role in the expression of *Dmrt2* or *Hmx2* genes in intercalated cells remains to be determined.

The exact mechanism mediating the downregulation of B-IC cells in cystic epithelia in TSC remains speculative. Our studies demonstrate a robust downregulation of *Hmx2*, which correlates with the loss of B-IC cells in cyst epithelia. One possible explanation for the predominance of A-IC and the absence of B-IC cells in TSC renal cysts is that the robust overexpression of DMRT2 in TSC models, while leading to AI-C cell preponderance, can inhibit HMX2 activity, thereby reducing B-IC cell numbers [21]. In addition, while the factors driving A-IC expansion in our system remain unclear, several pathways, such as the signaling axis involving the CXC chemokine receptor 4 and hensin, or molecules that mediate A-IC expansion (e.g., G-protein coupled receptor 4, CXC chemokine 12 or hypoxia-inducible factor 1α) could play critical roles in the increased A-IC and decreased B-IC cells lining the kidney cysts in TSC [40,41]. This observation is not at odds with the critical role of FOXP1 (and FOXI1) in the development and growth of intercalated cells (both A-IC and B-IC). Our results suggest that TSC kidney cystogenesis is driven by factors that exclusively promote A-IC cell proliferation while suppressing B-IC cells [16]. The mechanistic link between mTORC1 activity and altered expression of transcription factors that drive intercalated cell development and differentiation remains unclear. Our results show that the ablation of the *Foxi1* gene in Tsc1 KO mice prevents renal cyst formation and reduces the activity of mTORC1 [10]. Our RNA seq data also indicate that, while the ablation of the FOXI1 does not affect the expression of *Foxp1*, it significantly reduces the expression of both *Dmrt2* and *Hmx2* in both *Foxi1* KO and *Tsc1/Foxi1* dKO mice, indicating that they are downstream of FOXI1 (Figure 1A) as well as FOXP1 [16]. These results suggest that mTORC1 potentiation, while important for cyst formation, may depend on the complementary activation of IC and specifically A-IC development pathways. Given the known function of the Notch—HES1 pathway in promoting both A- and B-IC cells’ development and given the presence of both TSC1 and TSC2 proteins in cystic epithelia, we suggest that the de-differentiation of PC into intercalated cells may not be a major factor in enhancing A-IC cell development in the cystic epithelium of kidneys in TSC mouse models.

These studies clearly demonstrate the enhanced expression of established components of a TF network that drives A-IC cell differentiation in TSC renal cystic disease (FOXI1, FOXP1 and DMRT2). However, other than FOXI1, the functional role of these transcription factors in TSC renal cystogenesis is not specifically addressed. Identifying the specific molecules or pathways that enhance the expression of FOXI1, FOXP1 and DMRT2 driving A-IC cell predominance and downregulate HMX2, leading to the loss of B-IC, is another important aspect of TSC renal cystogenesis that needs to be addressed. Further studies as to the role of A-IC expansion and B-IC cell loss in TSC cystogenesis remain speculative.

## 4. Materials and Methods

### 4.1. Animal Models: Tsc1-KO, Tsc1/Car2 dKO, and Tsc2^+/−^ Mice

All mice used in this study were housed in a temperature (22 °C) and light controlled (12 h light/12 h dark) room and provided with food and water ad libitum. Studies were performed according to standards set forth by “The Guide for the Care and Use of Laboratory Animals” and were conducted under protocols approved by the Institutional Animal Care and Use Committee (IACUC) at the University of New Mexico (23-201353-HSC and 25-201691-B-HSC). All animal handlers were trained according to IACUC requirements. Animals were euthanized with an excess of pentobarbital sodium according to institutional guidelines and approved protocols.

### 4.2. Genotyping Protocols for Tsc1-KO, Tsc1/Car dKO, and Tsc2^+/−^ Mice

*Tsc1*-KO mice were generated by crossing *Tsc1^f/f^* mice [42] (Jackson Labs, #005680; Bar Harbor, ME, USA) with *Aqp2* Cre mice [43] (Jackson Labs, #006881). PCR was performed to identify mice of interest by isolating DNA from tail clips. The following primer sequences were used to identify the *Tsc1* conditional and null alleles: *Tsc1*: F4536 (5′-AGG AGG CCT CTT CTG CTA CC-3′), R4830 (5′-CAG CTC CGA CCA TGA AGT G-3′), and R6548 (5′-TGG GTC CTG ACC TAT CTC CTA-3′). PCR conditions for *Tsc1* were as follows: 94 °C for 5 min, 1 cycle; 94 °C for 30 s, 58 °C for 30 s, and 72 °C for 30 s, 45 cycles; 72 °C for 7 min, 1 cycle; and held at 4 °C. Combining all three oligonucleotides in the PCR provides amplification products of size 295 for a WT allele, 486 for a conditional allele, and 368 for a null allele [42]. In addition, PCR was conducted to identify the presence or absence of the *Aqp2* transgene utilizing the following oligonucleotides and PCR conditions: *mAqp-2* F (5′-CCT CTG CAG GAA CTG GTG CTG G-3′) and Cre-Tag R (5′-GCG AAC ATC TTC AGG TTC TGC GG-3′) run at 94 °C for 5 min, 1 cycle; 94 °C for 30 s, 63 °C for 30 s, and 72 °C for 1:30 min, 40 cycles; 72 °C for 7 min, 1 cycle; and held at 4 °C. The presence of the *Aqp-2* transgene resulted in an amplified 671-base-pair (bp) band signifying the junction between the mouse Aqp-2 promoter and the Cre gene.

*Tsc1*/carbonic anhydrase 2 double knockout (*Tsc1/Car2* dKO) mice were generated by crossing *Tsc1*-KO (*Tsc1/Aqp2* Cre) mice with *Car2* global KO mice. Genotyping for the *Car2* portion of these mice was performed utilizing DNA isolated from tail clips using the following oligonucleotides and PCR conditions: *Car2* Sense (5′-AAC CCC TGC ATT TCT GCA GAT TGG ACC TGC GTC A-3′) and *Car2* Antisense (5′-TGG AAG CAA TTA TTT ACC TCC GG-3′) run at 94 °C for 2 min, 1 cycle; 94 °C for 30 s, 62 °C for 30 s, and 68 °C for 1 min, 50 cycles; 68 °C for 1 min, 1 cycle; and held at 4 °C. The PCR products were then digested with Tsp45I (New England Biolabs, #R0583S; Ipswich, MA, USA) at 63° for 3 h and run on a 3% agarose gel, where a WT allele results in 162 and 34 bp bands, while a KO allele has only a 195 bp band [44].

Global *Tsc2^−/−^* are embryonically lethal [45]; therefore, *Tsc2^+/^^−^* mice were utilized for this study. *Tsc2^+/−^* mice present with multiple cysts and cystadenomas representing symptoms associated with tuberous sclerosis [45]. Genotyping for *Tsc2^+/−^* mice was performed utilizing DNA isolated from tail clips using the following oligonucleotides and PCR conditions: H162 (5′-CAAACCCACCTCCTCAAGCTTC-3′); H163 (5′-ATTGCGGCCTCAACAATCG-3′); and H164 (5′-AGACTGCCTTGGGAAAAGCG-3′). The following conditions were used for the PCR genotyping: 94 °C for 2 min, 1 cycle; 94 °C for 30 s, 58 °C for 30 s, and 72 °C for 1 min, 35 cycles; 72 °C for 5 min, 1 cycle; and held at 4 °C producing an 86 bp wild-type (WT) band and a 105 bp mutant band.

All animals used in these studies were bred onto a C57BL/6 background.

### 4.3. Tissue Collection

Mice were euthanized with an overdose of pentobarbital sodium and kidneys were quickly removed and placed in 4% paraformaldehyde in phosphate-buffered saline (PBS) at 4 °C for 24 h, then transferred to 70% ethanol. Tissues were then embedded in paraffin, and 5 μm sections were cut and put on slides for immunofluorescence and immunohistochemical studies. For future RNA isolation, kidneys were removed, snap frozen in liquid nitrogen and stored at −80 °C.

### 4.4. RNA-seq Analysis

RNA-seq analyses were performed on whole kidney RNA by Novogene Bioinformatics Technology Co., Ltd. (Beaverton, OR, USA). Briefly, mouse kidney tissues were harvested, snap frozen in liquid nitrogen, and stored at −80 °C. RNA isolation was performed using the TRI-reagent protocol (Molecular Research Center, #TR 118; Cincinnati, OH, USA). The isolated RNA samples were ethanol precipitated, dissolved in water, and subjected to quality control analysis using an Agilent 2100 Bioanalyzer with RNA 6000 Nano Kits (Agilent, Santa Clara, CA, USA). The redissolved samples were subjected to poly A selection, fragmented, and reverse-transcribed to generate complementary DNA libraries that were utilized for sequencing analysis. Libraries were sequenced using the HiSeqTM 2500 system (Illumina). Clean reads were aligned to the mouse refence genome using Hisat2 v2.0.4. Gene. The gene expression levels were determined using fragments per kilobase of transcript per million mapped fragments (FPKM) by HTSeq v0.9.1. Statistical analysis was performed by Novogene Bioinformatics Technology Co., Ltd. (Beaverton, OR, USA) using the original raw, integer-based read counts generated using HTSeq. The enrichment analysis of DET was performed using the ShinyGO application (http://bioinformatics.sdstate.edu/go/, accessed on 19 February 2024). The transcripts of interest in these studies were chosen based on the literature that described their role in the development of intercalated cells and differentiation of these cells to A-IC vs. B-IC cells [16,20,21]. The expression of the proteins coded by the transcripts identified in RNA-seq studies was characterized using Western blot analysis and localized using immunohistochemistry and immunofluorescence microscopy.

### 4.5. Immunofluorescence Microscopy

Slides were baked at 60 °C for 2 h and allowed to cool for 30 min then deparaffinized with xylene and rehydrated using decreasing percentages of ethanol. Next, slides underwent antigen retrieval utilizing a series of heating and cooling steps with 10 mM sodium citrate. Slides were then incubated in primary antibodies (see Table 1) in a humidity chamber overnight at 4 °C. The following day, slides were washed in phosphate-buffered saline (PBS, pH 7.4) and incubated in secondary antibodies (Alexa Fluor 594 goat-anti-rabbit 1:200 #A11037 and Alex Fluor 488 goat-anti-mouse 1:200 #A11029; ThermoFisher Scientific, Waltham, MA, USA) for 2 h at room temperature. Slides were again washed in PBS, allowed to air dry, and cover-slipped using Vectashield HardSet mounting media (Vector Laboratories #H-1500; Newark, CA, USA). Immunofluorescence images were obtained using a Zeiss LSM800 Airyscan microscope (Zeiss, Oberkochen, Germany) with Zeiss Zen software (Version 2.6).

### 4.6. Immunohistochemistry Microscopy

Slides utilized for immunohistochemistry underwent the same antigen retrieval procedure as immunofluorescence slides listed above. Following antigen retrieval, Bloxall blocking solution (Vector Labs, #SP-6000-100; Newark, CA, USA) was placed on the slides for 10 min at room temperature, followed by a wash in PBS and incubation in 10% normal goat serum. The primary antibody was added (see Table 1) and slides were incubated overnight at 4 °C in a humidity chamber. The next day, the slides were treated according to the manufacturer’s protocol for the Vectastain Elite ABC Kit (Vector Labs, #PK-6101) and later stained with the Vector VIP Substrate Kit (Vector Labs, #SK-4600). Slides were dried and cover-slipped with VectaMount Permanent Mounting Medium (Vector Labs, #H-5000). Images were obtained with a Zeiss Axio Imager.M2 microscope (Zeiss, Oberkochen, Germany).

### 4.7. Fluorescence In Situ Hybridization (FISH)

Paraffin-embedded tissue was prepared for staining with RNA-scope Multiplex Fluorescent V2 using probes Mm-Tsc1 (#591301), Mm-Tsc2 (#416461), and Mm-Foxi1 (#483021) (Advanced Cell Diagnostics, Newark, CA, USA). Sample preparation and labeling were performed according to the manufacturer’s protocol (Advanced Cell Diagnostics; Newark, CA, USA). TSA Vivid fluorophore 570 or 520 (Advanced Cell Diagnostics, #323272 and #323271; 1:1000) was used for visualization. Slides were air-dried and cover-slipped using Vectashield HardSet mounting media (Vector Laboratories #H-1500). Confocal imaging was carried out in the University of New Mexico Cancer Center Fluorescence Microscopy Shared Resource using an LSM 800 AiryScan confocal microscope (Zeiss, Oberkochen, Germany) and Zeiss Zen software (Version 2.6).

### 4.8. Western Blot Analysis

Protein was isolated from the kidneys of *Tsc1* KO (28 and 45 days old) and *Tsc1/Car2* dKO (47 and 105 days) mice in a solution of 10 mM triethanolamine and 250 mM sucrose adjusted to pH 7.6 with 1N NaOH. Tissues were homogenized for 30 s and allowed to cool on ice for 30 s (repeated 3 times). The homogenate was centrifuged at 4000× *g* at 4 °C for 10 min. The supernatant was removed, and protein concentration was measured utilizing a BCA Protein Assay kit (ThermoFisher #23225; Rockford, IL, USA). Samples were placed in Laemmli Buffer (BioRad #161-0737; Hercules, CA, USA) and β-mercaptoethanol, and heated at 95 °C for 10 min.

Denatured protein (30 μg in Laemmli buffer with 2-mercaptoethanol) was loaded onto a 4–12% Novex gel (ThermoFisher #XP04120BOX) with Tris-Glycine-SDS running buffer (Bio-Rad #161-0732) and run at 110 volts for 60 min. Afterwards, the gel was transferred onto a nitrocellulose membrane (ThermoFisher, #LC2001) and run at 25 volts for 90 min in Tris/Glycine transfer buffer (Bio-Rad #161-0734). The membrane was removed, washed, and incubated in 5% milk containing the antibody overnight at 4 °C.

The following day, membranes were washed and incubated in secondary antibody (donkey-anti-rabbit IgG; Thermofisher #31458) diluted in 5% milk. Membranes were washed and incubated in Novex ECL Chemiluminescence (ThermoFisher; #WP20005), and developed on autoradiography film (Alkali Scientific #XR1810; Ft. Lauderdale, FL, USA).

## Figures and Tables

**Table 1:** List of antibodies used in this manuscript.

Antibody	Vendor	Catalogue Number	Dilution
AQP2	Dr. Ann Blanchard (Université Paris Descartes, Paris, France)	N/A	1:5000
AQP2	Santa Cruz Biotechnologies	sc-515770	1:50
DMRT2	Novus Biologicals	NBP1-86361	1:75
FOXP1	Cell Signaling	4402	1:100 (IHC); 1:50 (IF)
H+-ATPase	Abcam	AB200839	1:200
H+-ATPase	Soleimani Lab	N/A	1:75
PCNA	Santa Cruz Biotechnologies	sc-56	1:50

## Data Availability

The original contributions presented in this study are included in the article/Supplementary Material. Further inquiries can be directed to the corresponding author.

## Supplementary Materials

The following supporting information can be downloaded at: https://www.mdpi.com/article/10.3390/ijms27177515/s1.

## Abbreviations

The following abbreviations are used in this manuscript:

ADPKD	Autosomal Dominant Polycystic Kidney Disease
A-IC	A-Intercalated Cells
AML	Angiomyolipomata
B-IC	B-Intercalated Cells
DMRT2	Doublesex and Mab-3-related Transcription Factors
FISH	Fluorescence In Situ Hybridization
FOXI1	Forkhead Box I1
mTORC1	Mechanistic Target of Rapamycin Complex 1
PC	Principal Cells
PCNA	Proliferating Cell Nuclear Antigen
pS6K	Phosphorylated Ribosomal S6 Protein
S6K	Phosphorylated Ribosomal S6 Kinase
TF	Transcription Factors
TSC	Tuberous Sclerosis Complex
