# Supplementary material for: Kidney Cyst Epithelia in Tuberous Sclerosis Complex (TSC) Exhibit Propagation and Expansion of A-Intercalated, but Loss of B-Intercalated and Principal Cells: The Role of FOXI1, FOXP1, and DMRT2 Transcription Factors
Source: Int J Mol Sci. 2026 Aug 22;27(17):7515. doi: 10.3390/ijms27177515 (PMC13565657; doi:10.3390/ijms27177515)
Supplement: Supplementary file 1 [file ijms-27-07515-s001.zip › ijms-4432253-supplementary.pdf]

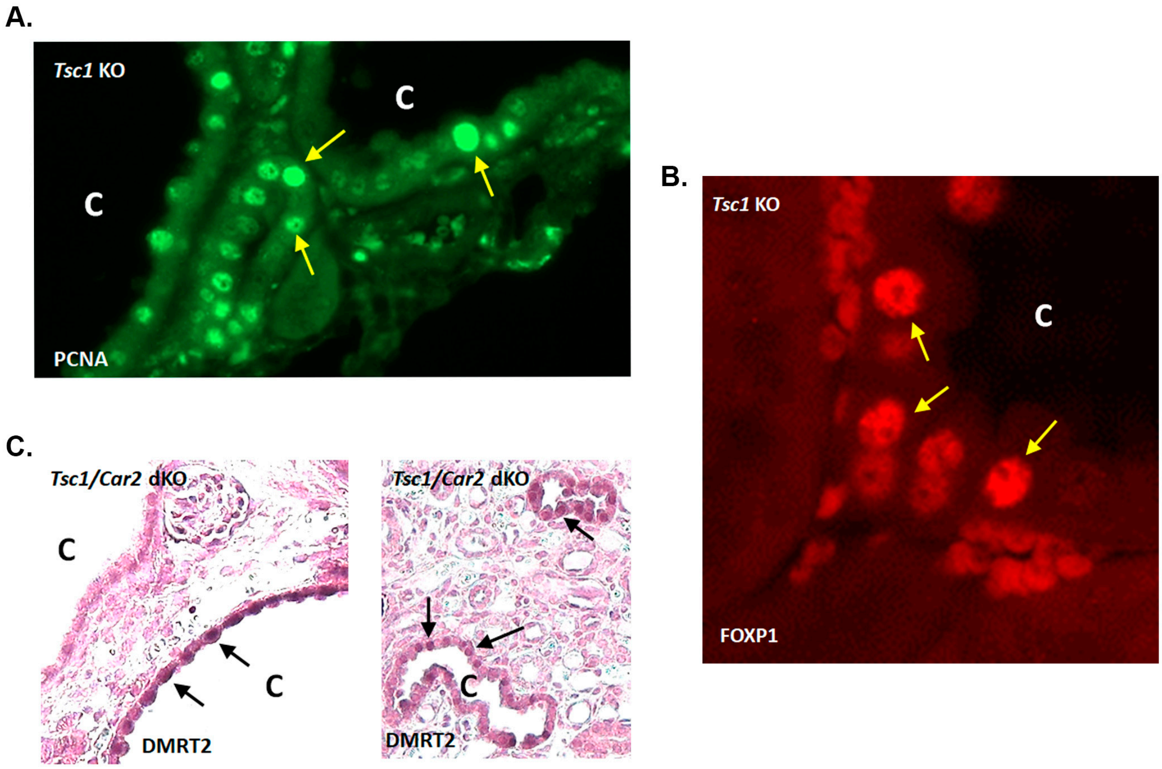

Figure S1. High magnification images showing nuclear localization of PCNA and FOXP1. Enlarged magnification depicting the nuclear localization (yellow arrows) of (A) PCNA from Figure 3A and (B) FOXP1 from Figure 6B. Supplement Figure S1C shows an enlargement depicting DMRT2 nuclear staining lining the cystic epithelium in Tsc1/Car2 dKO mice.
